# Personal contact with HIV-positive persons is associated with reduced HIV-related stigma: cross-sectional analysis of general population surveys from 26 countries in sub-Saharan Africa

**DOI:** 10.7448/IAS.20.1.21395

**Published:** 2017-01-10

**Authors:** Brian T Chan, Alexander C Tsai

**Affiliations:** ^a^ Division of Infectious Diseases, Brigham and Women’s Hospital, Boston, MA, USA; ^b^ Harvard Medical School, Boston, MA, USA; ^c^ Chester M. Pierce, MD Division of Global Psychiatry, Massachusetts General Hospital, Boston, MA, USA; ^d^ Mbarara University of Science and Technology, Mbarara, Uganda

**Keywords:** Stigma, HIV, Africa, contact hypothesis, social distancing

## Abstract

**Introduction**: HIV-related stigma hampers treatment and prevention efforts worldwide. Effective interventions to counter HIV-related stigma are greatly needed. Although the “contact hypothesis” suggests that personal contact with persons living with HIV (PLHIV) may reduce stigmatizing attitudes in the general population, empirical evidence in support of this hypothesis is lacking. Our aim was to estimate the association between personal contact with PLHIV and HIV-related stigma among the general population of sub-Saharan Africa.

**Methods**: *Social distance* and *anticipated stigma* were operationalized using standard HIV-related stigma questions contained in the Demographic and Health Surveys and AIDS Indicator Surveys of 26 African countries between 2003 and 2008. We fitted multivariable logistic regression models with country-level fixed effects, specifying social distance as the dependent variable and personal contact with PLHIV as the primary explanatory variable of interest.

**Results**: We analyzed data from 206,717 women and 91,549 men living in 26 sub-Saharan African countries. We estimated a statistically significant negative association between personal contact with PLHIV and desires for social distance (adjusted odds ratio [AOR] = 0.80; *p* < 0.001; 95% Confidence Interval [CI], 0.73–0.88). In a sensitivity analysis, a similar finding was obtained with a model that used a community-level variable for personal contact with PLHIV (AOR = 0.92; *p* < 0.001; 95% CI, 0.89–0.95).

**Conclusions**: Personal contact with PLHIV was associated with reduced desires for social distance among the general population of sub-Saharan Africa. More contact interventions should be developed and tested to reduce the stigma of HIV.

## Introduction

HIV-related stigma – the social discrediting or devaluation associated with HIV [1] – has been identified as a critical impediment to HIV prevention and treatment efforts worldwide [2,3], given its association with reduced uptake of voluntary counselling and testing [4,5], increased sexual risk-taking behaviour [6,7], reduced likelihood of serostatus disclosure [8,9], and poorer adherence to antiretroviral therapy (ART) [10,11]. Dimensions of HIV-related stigma in the general population include *negative attitudes* towards people living with HIV (PLHIV), including *desires for social distance* [12], that may be manifested behaviourally (*enacted stigma* targeting PLHIV through either word or action [13,14]). Furthermore, persons in the general population may experience *anticipated stigma*. Although this term has been most commonly applied to persons with a stigmatized attribute [15] in reference to the expectation of negative consequences such as rejection or condemnation due to their having the stigmatized attribute [12], anticipated stigma can also be assessed among the general population as the expectation of negative consequences that would result if one’s *hypothetical* HIV infection were disclosed to others [16–19].

It has been theorized that ART scale-up may counter HIV-related stigma by weakening the associations between HIV and economic incapacity, social exclusion, and imminent death [20–22]. Nevertheless, despite the expansion of ART in sub-Saharan Africa in the 21st century, HIV-related stigma in the general population remains highly prevalent [16,18]. Unfortunately, policymakers have available relatively few evidence-informed interventions proven to substantially reduce stigma on either an individual or population-based level [23–25].

Further development and refinement of anti-stigma interventions will therefore be crucial for the achievement of HIV prevention and treatment targets in sub-Saharan Africa and around the world. One approach that holds promise is directly involving PLHIV in the development and implementation of anti-stigma interventions. The mechanism by which interventions involving PLHIV may reduce stigma is summarized by the “contact hypothesis”. Originally put forth by Allport [26], the contact hypothesis suggests that discriminatory attitudes towards groups seen as the “other” may be reduced by interpersonal interactions if facilitated under certain conditions [27,28]. Such interactions are purported to lead to greater knowledge and reduced stereotyping of members of the stigmatized group [27], in turn lessening fear and prejudice [29]. In the literature on mental-illness-related stigma, contact interventions have been found to be effective in reducing stigma in general population samples, at least in the short term and particularly in high-income settings [30].

In the HIV context, it has been theorized that personal contact with PLHIV, especially PLHIV who have benefited from the salubrious effects of ART [21,31], should result in decreased fear, misunderstanding and branding of PLHIV as the “other” [32,33]. Contact interventions to reduce HIV-related stigma among health care professionals have been trialled in multiple low- and middle-income countries (LMICs), including one study conducted in five African countries [34] as well as examples from China [35,36], Thailand [37] and India [38]. Furthermore, direct contact with PLHIV as part of a multi-pronged intervention to reduce stigma in the general population has been studied in countries including Thailand [39] and Vietnam [40].

However, there is limited evidence to support the hypothesis that personal contact with PLHIV reduces HIV-related stigma. In a 1990–1992 cross-sectional study conducted in the United States, survey respondents who had an history of direct contact with PLHIV were less likely to support coercive policies targeting PLHIV, hold blaming attitudes towards PLHIV, and report avoidance of PLHIV [41]. These findings were confirmed in a more recent sample of religious congregants in the United States [42]. In studies conducted outside the US, contact with PLHIV was associated with decreased stigma and discriminatory attitudes among health providers [43] and employees of non-governmental organizations [44] in India, as well as in a general community sample in South Africa [45]. There have been several contrasting findings. For example, analyzing data from the South African Cape Area Panel Survey, Maughan-Brown [46] did not find an association between contacts with PLHIV and decreased HIV-related stigma. Notably, other than the two South African studies – one of which yielded a null result – no studies have been conducted in general population samples in LMICs or, most importantly, since the advent of widespread ART. Chan and Tsai [16] analyzed data from 31 sub-Saharan African countries and found that ART scale-up was associated with declines in desires for social distance in the general population; this effect was more pronounced in countries with relatively high HIV prevalence. That analysis provided indirect evidence that personal contact with PLHIV on ART might diminish the links between HIV and economic incapacity and social death, leading to a subsequent decline in social distancing.

Understanding the extent to which personal contact with PLHIV is associated with reduced HIV-related stigma in the general population of sub-Saharan Africa is important for policymakers, as finding a strong association would support the development and testing of anti-stigma interventions that prominently involve PLHIV. To help answer this question, we analyzed cross-sectional, individual-level data pooled from the Demographic and Health Surveys (DHS) and AIDS Indicator Surveys (AIS). Our primary aim was to estimate the association between personal contact with PLHIV and either *desires for social distance* or *anticipated stigma*, using data from general population samples in sub-Saharan Africa during a period of ART scale-up.

## Methods

The DHS and AIS are nationally representative, population-based surveys conducted approximately every five years in over 90 LMICs [47]. The standardization of DHS/AIS questions, including those on HIV-related stigma, allows for the analysis of temporal trends in attitudes and behaviours within countries [16,17] as well as comparative analyses across countries [18,48]. Details of the DHS/AIS sampling procedures are available on the DHS website and in reports published for each country [49]. We pooled individual-level data from 26 DHS/AIS conducted in countries in sub-Saharan Africa between 2003 and 2008 into a single dataset, using a de-normalization procedure to take into account the survey weights for each country-level dataset [47]. This time frame was chosen because this was a period of increasing ART availability and because the DHS/AIS measure for personal contact with PLHIV (described below) was largely phased out after 2008.

This dataset was then merged with country-level data on HIV prevalence from the UNAIDS AIDSInfo online database [50]. UNAIDS estimates country HIV prevalence using a modelling approach that incorporates data from antenatal clinics and nationally representative population-based surveys that include blood testing [51,52]. For cases in which the DHS data spanned two years (e.g. 2003–2004), we abstracted country HIV prevalence from the first year of the survey. There were five countries with a DHS survey in 2003 (Ghana, Kenya, Madagascar, Mozambique and Nigeria), but UNAIDS data on HIV prevalence were not available prior to 2004. For these countries, we matched the UNAIDS data from 2004 with the DHS from 2003. Ethical approval for each DHS/AIS survey was obtained from appropriate national entities; all data used for this analysis are de-identified and publicly available [49].

### Measures

The primary outcomes of interest were *desires for social distance* and *anticipated stigma*. The DHS/AIS include three questions which measure desires for social distance: (1) “If a member of your family became sick with AIDS, would you be willing to care for her or him in your own household?”; (2) “Would you buy fresh vegetables from a shopkeeper or vendor if you knew that this person had the AIDS virus?”; and (3) “In your opinion, if a female teacher has the AIDS virus but is not sick, should she be allowed to continue teaching in the school?” Negative responses to these questions reflect expressions of social distance [12], often motivated by instrumental concerns about casual transmission of HIV or preoccupations with the symbolic association of HIV with perceived deviance [53]. We defined a respondent as having a desire for social distance if he or she had a negative response to at least one of these three questions. The DHS/AIS include one question on anticipated stigma applicable to a general population sample, “If a member of your family got infected with the AIDS virus, would you want it to remain a secret or not?” Positive responses to this question reflect fear of disclosing a hypothetical HIV infection [54], in particular the expectation of rejection or condemnation were a family member’s serostatus revealed to others [55].

The primary exposure of interest was *personal contact with PLHIV*, which was ascertained by one question, “Do you personally know someone who is suspected to have the AIDS virus or who has the AIDS virus?” Because of the possibility of reverse causality, in that persons without desires for social distance towards PLHIV may be more willing to maintain relationships with PLHIV (or admit that they know PLHIV), we also created a community-level summary variable representing the percentage of participants in a primary sampling unit (PSU) reporting personal contact with PLHIV (exclusive of the index participant). In the DHS/AIS, the PSU is the smallest clustering unit of analysis, typically a village in rural areas and a ward or residential neighbourhood in urban areas. In the remainder of the manuscript, we refer to this level of analysis as the “village” for ease of exposition. Villages with fewer than five participants were removed from the analysis.

Socio-demographic variables (age, gender, educational attainment, marital status, household asset wealth [56,57] and employment status), year of DHS/AIS, an HIV knowledge variable equal to the number of correct responses to six questions about HIV prevention and transmission (see Additional File 1), and country HIV prevalence were included in the regression models as potential confounders of the relationship between personal contact with PLHIV and stigma.

### Statistical analysis

We used descriptive statistics to characterize the sample, including t-tests or chi-square tests for differences by gender. For the primary analyses, we fitted multivariable logistic regression models with cluster-correlated robust standard errors [58–60] and country-level fixed effects, alternately specifying social distance or anticipated stigma as the dependent variable, and personal contact with PLHIV as the primary exposure of interest. A statistically significant regression coefficient was considered evidence that an association existed between HIV-related stigma and personal contact with PLHIV. We then fitted multivariable regression models to the data from each country sample separately. As a sensitivity analysis, we fitted multivariable-ordered logistic regression models with an ordinal composite variable for individual-level social distance, with values ranging from zero (answering no to all three questions) to three (answering yes to all three questions), as the outcome of interest.

Of note, the observed association between personal contact and HIV stigma could result from reverse causality. For example, persons who do not hold stigmatizing attitudes towards PLHIV may be more willing to be in relationships with PLHIV. To address this possibility, in another sensitivity analysis, we fitted multivariable logistic regression models using the percentage of respondents in the study participant’s village reporting personal contact with PLHIV as the exposure of interest [4,61,62]. All analyses were performed using Stata software (Version 13.1, StataCorp, College Station, TX, USA).

## Results

### Study population

206,717 women and 91,549 men from 26 sub-Saharan African countries with complete data for the variables of interest were included in the analyses. Survey refusal rates among men and women in the DHS/AIS were typically less than 10%, and no survey had a refusal rate exceeding 20%. DHS/AIS respondent characteristics are stratified by gender in Table 1.

**Table 1.** Characteristics of DHS/AIS participants from 26 sub-Saharan African countries, by gender

Characteristic	Overall (*n* = 298,266)	Women (*n* = 206,717)	Men (*n* = 91,549)
Age, mean (SD), y	29.0 (10.2)	28.3 (9.4)	30.5 (11.6)
Achieved more than primary education	33%	29%	43%
Married	62%	65%	55%
Household asset index, mean (SD)*	14,416 (109,326)	15,254 (109,990)	12,526 (107,791)
Employed	63%	60%	69%
Knows someone infected with HIV	36%	35%	37%
Endorsed desire for social distance	62%	65%	56%
Endorsed anticipated stigma	44%	47%	38%

DHS, Demographic and Health Surveys. AIS, AIDS Indicator Surveys. y, year. SD, standard deviation. SE, standard error. All t-tests /chi-square tests for differences by gender yielded *p*-values of less than 0.001. *More information about the construction of the household asset index can be found in Filmer & Pritchett (1999, 2001). Information about how the household asset index was specifically operationalized in the DHS/AIS is available at: http://www.dhsprogram.com/topics/wealth-index/Index.cfm

### HIV-related stigma and contact with PLHIV

Across all surveys, 62% of respondents endorsed at least one measure of social distance, while 44% endorsed anticipated stigma. The scale reliability coefficient for the three social distancing questions was 0.61. Although it is difficult to interpret *p*-values in light of the large sample size, women appeared more likely to endorse desires for social distancing and anticipated stigma. Women were only slightly less likely to have had personal contact with PLHIV.

### Regression analyses

**Figure 1.**
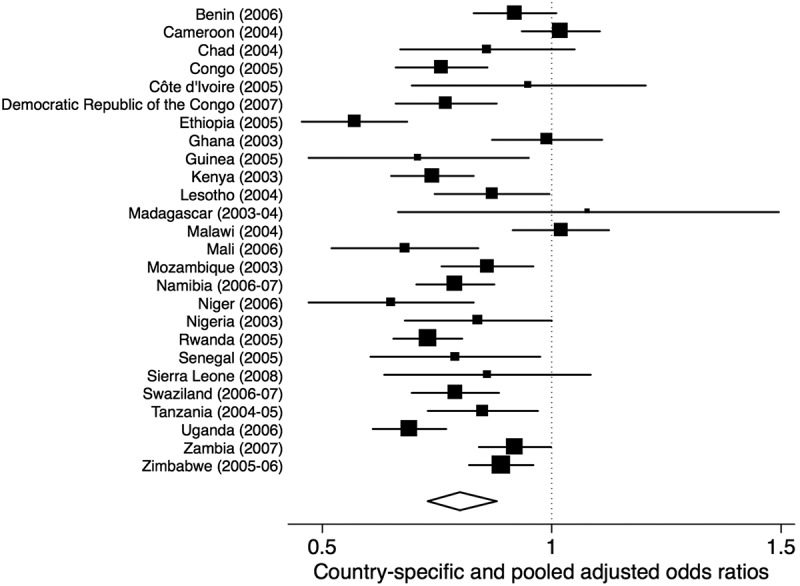
Forest plot of country-specific estimates for the association between personal contact with PLHIV and desires for social distance.

In a multivariable regression model fitted to the pooled data (Table 2), we estimated a statistically significant negative association between personal contact with PLHIV and desires for social distance (adjusted odds ratio [AOR] = 0.80; *p* < 0.001; 95% Confidence Interval [CI], 0.73–0.88). Evaluated at the mean of the other covariates, a history of personal contact with PLHIV was associated with a 4% absolute decrease in the predicted probability of a desire for social distance, from 69% to 65%. In the country-specific analyses, the adjusted odds ratios for the association between personal contact with PLHIV and desires for social distance were less than one in 23 of 26 countries, and of these 15 were statistically significant (Figure 1; Additional File 2). The sensitivity analysis using an ordinal composite variable for social distancing yielded similar findings (AOR = 0.75; *p* < 0.001; 95% CI, 0.69–0.82) compared with the binary outcome.

**Table 2.** Unadjusted and adjusted odds ratios and 95% confidence intervals for variables associated with desires for social distance in the general population

Variable	Unadjusted odds ratio* (95% CI)	Adjusted odds ratio (95% CI)
Personal contact with PLHIV	0.612 (0.514–0.729)	0.798 (0.726–0.876)
Female	1.377 (1.243–1.524)	1.164 (1.064–1.272)
Age (per year)	0.999 (0.993–1.004)	0.992 (0.989–0.996)
Achieved secondary education or higher	0.267 (0.210–0.340)	0.522 (0.429–0.635)
Married	1.265 (1.095–1.461)	1.063 (0.995–1.136)
Household asset index (divided by 10000) **	0.947 (0.938–0.955)	0.969 (0.963–0.975)
Employed	1.060 (0.985–1.141)	1.007 (0.971–1.045)
DHS/AIS (per year)	0.941 (0.936–0.946)	0.514 (0.499–0.530)
HIV knowledge (per correctly answered question, out of 6)	0.601 (0.558–0.647)	0.685 (0.644–0.729)
Country HIV prevalence (each per cent increase)	0.957 (0.957–0.957)	0.949 (0.946–0.953)

CI, confidence interval. * Adjusted for country-level fixed effects only, where appropriate ** More information about the construction of the household asset index can be found in Filmer & Pritchett (1999, 2001). Information about how the household asset index was specifically operationalized in the DHS/AIS is available at: http://www.dhsprogram.com/topics/wealth-index/Index.cfm

Turning next to the sensitivity analysis that used the village-level summary variable for personal contact with PLHIV, we found that study participants who lived in villages where a greater percentage of people reported knowing PLHIV were themselves less likely to endorse HIV-related stigma. For every 10% increase in the percentage of people in the village who reported knowing someone with HIV, there was an 8% reduced odds of social distancing (AOR = 0.92; *p* < 0.001; 95% CI, 0.89–0.95). Evaluated at the mean of the other covariates, study participants who lived in a village where 10% of respondents reported knowing PLHIV (25th percentile across villages) had a 71% predicted probability of endorsing a desire for social distance, whereas study participants who lived in a village where 58% of respondents reported knowing PLHIV (75th percentile across villages) had a 64% predicted probability of endorsing a desire for social distance.

In contrast to the findings about social distance, there was no apparent association with anticipated stigma. In multivariable regression models, we did not estimate a statistically significant association between personal contact with PLHIV and anticipated stigma using either the individual-level (AOR = 0.99; *p* = 0.69; 95% CI, 0.92–1.05) or village-level personal contact variable (AOR = 1.01; *p* = 0.33; 95% CI, 0.98–1.03).

## Discussion

In this cross-country analysis of data from 298,266 persons living in 26 sub-Saharan African countries, we found evidence for an association between personal contact with PLHIV and reduced desires for social distance in the general population. Our findings provide evidence in support of the “contact hypothesis”, which suggests that having personal contact with a member of a stigmatized group results in decreased fear, misunderstanding, and prejudice [27,28]. This association was statistically significant, robust to statistical adjustment by socio-demographic variables, year of DHS/AIS, HIV knowledge, and country HIV prevalence, and consistently estimated in most of the 26 countries under study. Although it is possible that persons who do not hold stigmatizing attitudes towards PLHIV may be more willing to maintain (or admit) relationships with PLHIV, this appeal to reverse causality is unlikely to completely explain our findings, given that an association was found using both an individual-level and village-level variable for personal contact with PLHIV.

Our findings have important implications for policymakers as they suggest a possible mechanism for enhancing interventions to reduce negative attitudes towards PLHIV in sub-Saharan Africa. To date, there remains a relative paucity of interventions proven to effect sustained reductions in HIV-related stigma on an individual or population-based level [23–25]. Ensuring that PLHIV participate in intervention development and implementation, thereby increasing opportunities for meaningful interactions between PLHIV and other members of the general population, may enhance the efficacy of anti-stigma interventions [43]. Several examples of interventions that prominently feature PLHIV have been attempted with some success in LMICs [34–40]. Additionally, our findings suggest an additional benefit to the judicious disclosure of serostatus; however, internalized stigma has been shown to inhibit disclosure [9] and there is only limited evidence to support the efficacy of interventions designed to encourage such disclosures [63].

Although we found an association between personal contact with PLHIV and reduced desires for social distancing, we did not find a similar association between personal contact with PLHIV and anticipated stigma in the general population. What could explain these divergent findings? One plausible explanation consistent with the contact hypothesis is that although personal contact with PLHIV would be expected to reduce desires for social distancing held by respondents, it would not change their beliefs that *other* people continue to hold negative attitudes towards PLHIV. Thus, even if one’s personal attitudes had changed, one could still harbour persistent fears of serostatus disclosure in the event of an hypothetical HIV infection.

There are several limitations to our study. First, our measures of social distance and anticipated stigma are self-reports of hypothetical scenarios that could be misconstrued by respondents [64–66], and our measure of personal contact with PLHIV uses an outdated term, “AIDS virus”. In addition, we use a single binary measure for anticipated stigma, rather than a scale. However, research from Tanzania suggests that most respondents held a reasonable understanding of these measures, including the binary anticipated stigma measure [64]. Other than the use of the term “AIDS virus”, these measures are similar to those that have been used by others [42,45,46]. Moreover, this limitation would only bias our estimates if the extent of misinterpretation systematically differed according to whether someone had personal contact with PLHIV, a scenario that we believe to be unlikely. Of note, the DHS is planning to revise the stigma indicators in future questionnaires, which may enhance their reliability and validity [67]. Second, our study did not include data from South Africa, the country with the world’s largest HIV epidemic. Nevertheless, our study is the most comprehensive analysis of this topic to date, including 26 countries and more than 200,000 persons. Third, our datasets are from 2003 to 2008 and therefore may not reflect the most current situation in sub-Saharan Africa. However, the most pertinent change from 2003–2008 to the present is the increasing availability of relatively simple, effective, and well-tolerated ART regimens in sub-Saharan Africa. Therefore, we believe it is even more likely in the present day that personal contact with PLHIV benefiting from ART should help to weaken links between HIV and economic incapacity, social exclusion, and inevitable death, leading to reduced fear and prejudice in the general population. Finally, although we have shown an association between personal contact with PLHIV and decreased social distancing, we cannot prove that the association is causal. Although, as stated previously, reverse causality is an unlikely explanation for our findings (e.g. it is implausible that one’s personal beliefs could influence the village-wide percentage of other persons who have had contact with PLHIV), it is possible that people who do not hold stigmatizing beliefs might be more willing to live in a village where there are more PLHIV or more people who know PLHIV. Conversely, it is possible that people who hold more stigmatizing beliefs might be less willing to live in a village with more PLHIV. Such a phenomenon would be consistent with the “white flight” phenomenon observed in high-income countries [68–70]. Nevertheless, we believe it is unlikely that this scenario would entirely account for the association that was found.

In conclusion, in this cross-country analysis of data from 26 countries in sub-Saharan Africa, we found that personal contact with PLHIV was associated with reduced desires for social distancing towards PLHIV in the general population. Our findings suggest that interventions that target HIV-related stigma may benefit from the prominent involvement of PLHIV to reduce fear, misunderstanding, and prejudice among the general population. This is highly relevant for policymakers given the pressing need for effective anti-stigma interventions to enhance HIV prevention and treatment initiatives. Further study is needed to develop and empirically test the efficacy of such interventions in sub-Saharan Africa and other LMICs.

## Notes

To access the supplementary material to this article please see Supplementary Files under Article Tools online.
